# High Endogenous Accumulation of ω-3 Polyunsaturated Fatty Acids Protect against Ischemia-Reperfusion Renal Injury through AMPK-Mediated Autophagy in Fat-1 Mice

**DOI:** 10.3390/ijms18102081

**Published:** 2017-09-30

**Authors:** Do Hyeong Gwon, Tae Woong Hwang, Ju-Ye Ro, Yoon-Joong Kang, Jin Young Jeong, Do-Kyung Kim, Kyu Lim, Dong Woon Kim, Dae Eun Choi, Jwa-Jin Kim

**Affiliations:** 1 Department of Anatomy, School of Medicine, Chungnam National University, Daejeon 35015, Korea; dohyeong171@gmail.com (D.H.G.); dkdnwl1234@gmail.com (T.W.H.); visnu528@cnu.ac.kr (D.W.K.); 2 Department of Medical Science, School of Medicine, Chungnam National University, Daejeon 35015, Korea; spwlsdud@naver.com; 3 Brain Research Institute, School of Medicine, Chungnam National University, Daejeon 35015, Korea; chamas89@gmail.com; 4 Department of Biomedical Science, Jungwon University, Geosan, Chungbuk 28023, Korea; yjkang@jwu.ac.kr; 5 Department of Nephrology, School of Medicine, Chungnam National University, Daejeon 35015, Korea; 6 Department of Anatomy, College of Medicine, Konyang University, Daejeon 35365, Korea; dokyung@konyang.ac.kr; 7 Department of Biochemistry, School of Medicine, Chungnam National University, Daejeon 35015, Korea; kyulim@cnu.ac.kr

**Keywords:** ischemia-reperfusion injury (IRI), fat-1 transgenic mice, AMP-activated protein kinase (AMPK), autophagy

## Abstract

Regulated autophagy is involved in the repair of renal ischemia-reperfusion injury (IRI). Fat-1 transgenic mice produce ω3-Polyunsaturated fatty acids (ω3-PUFAs) from ω6-Polyunsaturated fatty acids (ω6-PUFAs) without a dietary ω3-PUFAs supplement, leading to a high accumulation of omega-3 in various tissues. ω3-PUFAs show protective effects against various renal injuries and it has recently been reported that ω3-PUFAs regulate autophagy. We assessed whether ω3-PUFAs attenuated IR-induced acute kidney injury (AKI) and evaluated its associated mechanisms. C57Bl/6 background fat-1 mice and wild-type mice (wt) were divided into four groups: wt sham (*n* = 10), fat-1 sham (*n* = 10), wt IRI (reperfusion 35 min after clamping both the renal artery and vein; *n* = 15), and fat-1 IRI (*n* = 15). Kidneys and blood were harvested 24 h after IRI and renal histological and molecular data were collected. The kidneys of fat-1 mice showed better renal cell survival, renal function, and pathological damage than those of wt mice after IRI. In addition, fat-1 mice showed less oxidative stress and autophagy impairment; greater amounts of microtubule-associated protein 1A/1B-light chain 3 (LC3)-II, Beclin-1, and Atg7; lower amounts of p62; and, higher levels of renal cathepsin D and ATP6E than wt kidneys. They also showed more adenosine monophosphate-activated protein kinase (AMPK) activation, which resulted in the inhibition of phosphorylation of the mammalian target of rapamycin (mTOR). Collectively, ω3-PUFAs in fat-1 mice contributed to AMPK mediated autophagy activation, leading to a renoprotective response.

## 1. Introduction

Although there are many causes of acute renal dysfunction, renal ischemic and/or reperfusion injuries are a major cause of acute kidney injury [1]. Complex mechanisms are involved in ischemia-reperfusion injury (IRI). Hypoxic injury triggers the tubular cell secretion of cytokines associated with acute inflammatory processes, the generation of reactive oxygen species (ROS), and apoptotic and necrotic cell death [2,3,4]. Moreover, reperfusion following ischemia generates large amounts of ROS, resulting in tubular cell death [5]. ROS increases during ischemia reperfusion (IR) injury exerting toxic effects, including kidneys, and recent studies have shown that oxidative stress and associated autophagy signaling play important roles in renal IRI [6,7]. Recent reports have suggested that the activation of autophagy attenuates IRI of the kidney [8]. Mice with Autophagy-Related Protein 5 (*Atg5*) deletion in both proximal and distal tubules when subjected to IR injury also had more severe tubular damage and renal dysfunction, with decreased renal function after IR injury, and also accumulated damaged mitochondria and displayed an increased apoptosis (increase of cleaved caspase-3) [9].

Autophagy has recently been found to be an important regulator in adaptive cellular and tissue responses to IRI [8]. In general, autophagy is a natural homeostatic process responsible for removing misfolded proteins, damaged organelles, and intracellular pathogens, and plays a central role in maintaining survival mechanisms [7]. Autophagy can also be increased by non-nutrient dependent mechanisms during reperfusion via a process associated with ROS [10].

Many regulators of autophagy have been implicated, including Autophagy-Related Protein 1 (Atg1), the mammalian target of rapamycin (mTOR), and AMP-activated protein kinase (AMPK) [11]. mTOR, as a key regulator of autophagy, regulates the balance between cell growth and autophagy in response to nutritional status, growth factor, and stress signals, and interacts with regulatory proteins to form the mTOR complex 1 (mTORC1). mTORC1 functions as a nutrient sensor, which when activated, stimulates protein synthesis and cell growth, and blocks autophagy [12]. AMPK, in particular, is considered as a potentially important regulator of autophagy in the kidney [13]. Decreased nutrients and increased hypoxia increase AMPK and autophagy during ischemia [8,9], and the role of activated AMPK appears to be essential in maintaining intracellular redox status by inhibiting oxidant production by nicotinamide adenine dinucleotide phospate (NADPH) oxidases, including p22phox, or by increasing the expression of antioxidant enzymes such as UCP (uncoupling protein)-2 [14,15]. It is rapidly phosphorylated in ischemic tubular cells [16]. It has also been reported that 5-aminoimidazole-4-carboxamide ribonucleotide (AICAR, an AMPK agonist) is increased in microtubule-associated protein 1A/1B-light chain 3 (LC3) puncta, a marker of autophagy, resulting in attenuated renal IRI [16]. LC3, a mammalian homolog of yeast Atg8, is tightly associated with autophagosomal membranes. Thus, the measurement of LC3-II is regarded as a hallmark of autophagy activity [17]. The protein is itself degraded by autophagy, and cytoplasmic LC3-II protein is recruited to the autophagosomal membranes. The ubiquitin-associated protein p62 binds Atg8/LC3, and itself is degraded by autophagy. Since it accumulates when autophagy is inhibited, it also can be used to measure autophagic degradation [18,19].

ω3-Polyunsaturated fatty acids (ω3-PUFAs) have shown protective effects on various organ injuries, including cardiovascular diseases, liver, and brain injury models; improving depression and lipid profiles [20,21,22,23]. Additionally, ω3-PUFAs can also protect against ischemic injury in the kidney [24]. Indeed, significant concentrations of ω3-PUFAs are present in many human tissues [25], and are significantly involved in reducing ROS and attenuating ischemic acute renal failure [26]. Furthermore, ω3-PUFAs increase the phosphorylation of AMPK in ischemic intestines [27]. The energy-sensing LKB1-AMPK pathway moderates cell survival under a lack of energy, increasing the AMP:ATP ratio [28]. Moreover, AMPK has been implicated in many aspects of cell proliferation, apoptosis, and autophagy [29,30,31]. Furthermore, ω3-PUFAs are involved in modulating autophagy via Akt-mTOR signaling in prostate cancer and lung cancer [31,32]. Therefore, these reports suggest that ω3-PUFAs may regulate autophagy activation via the AMPK pathway in IR-induced kidneys.

Fat-1 transgenic mice express ω3 desaturase (fat-1) from Caenorhabditis elegans, leading to an increase of ω3-PUFAs from ω6-PUFAs. Fat-1 mice not only showed enhanced concentrations of n-3 α-linolenic acid (ALA), eicosapentaenoic acid (EPA), docosahexaenoic acid (DHA), and docosapentaenoic acid (DPA), but also significantly reduced n-6 linoleic and arachidonic acids in tissues including muscle, red blood cells, kidney, lung, spleen, heart, brain, and liver [33]. Despite this marked change in the ratio of n-6 to n-3, such transgenic mice are apparently normal and healthy. Thus, this model is ideal for studying the effects of the tissue n-6/n-3 ratio in the body.

## 2. Results

### 2.1. Effects of Endogenous ω3-Polyunsaturated Fatty Acids on Renal Function

**Figure 1:**
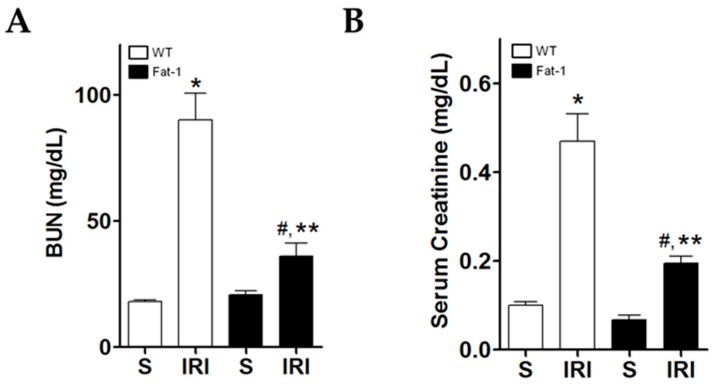
Renal function (WT-S, wild type sham; WT-IRI, ischemic-reperfusion renal injury (IRI) in wild-type mice; fat-1, fat-1 induction sham; fat-1 ischemia-reperfusion injury (IRI), ischemia-reperfusion (IR) renal injury in fat-1 induction mice). The levels of (**A**) blood urea nitrogen (BUN); and (**B**) serum creatinine were significantly increased in wt IRI mice when compared to sham mice. Fat-1 IRI mice showed decreased the levels of BUN and serum creatinine, compared to wt IRI mice. * *p* < 0.05 vs. WT sham kidney, # *p* < 0.05 vs. fat-1 sham kidney, ** *p* < 0.05 vs. WT IRI kidney. Bar represents mean ± S.D.

Blood urea nitrogen (BUN) and serum creatinine were measured to evaluate renal function. Levels of BUN and serum creatinine were significantly higher in wt IRI mice than in wt sham mice (Figure 1A,B). They were also significantly higher in fat-1 IRI mice than in fat-1 sham mice, and in wt IRI mice than in fat-1 IRI mice.

### 2.2. Effects of Endogenous ω3-Polyunsaturated Fatty Acids on Renal Tissue Injury

**Figure 2:**
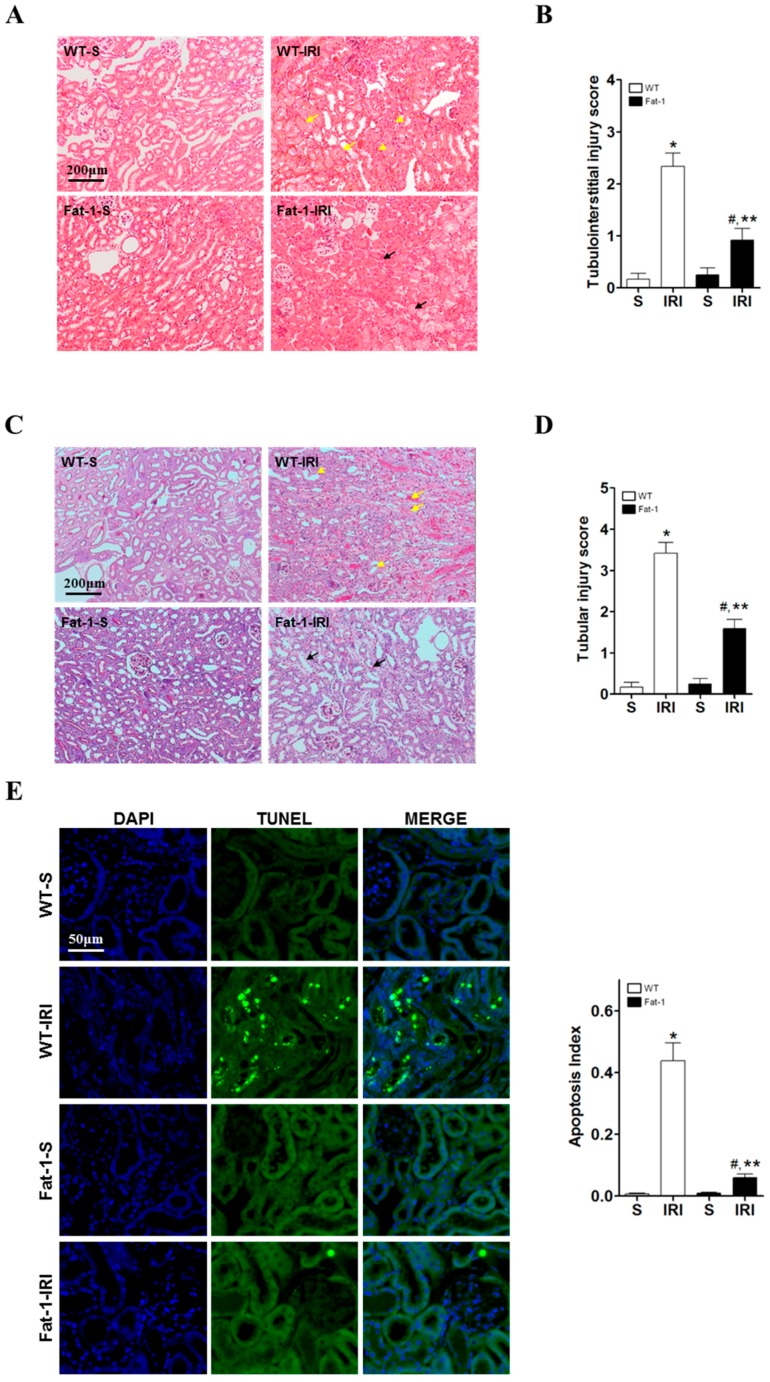

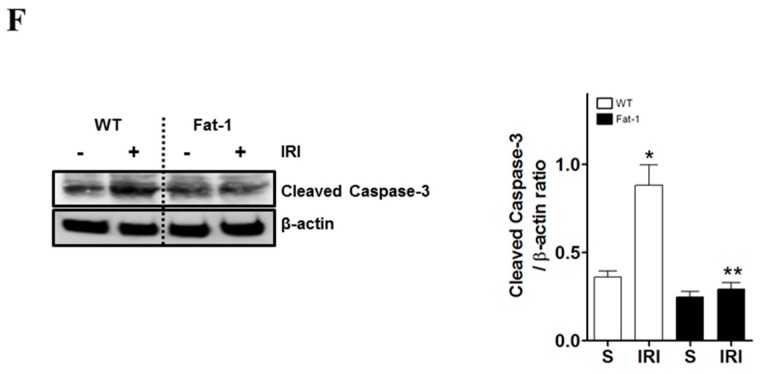
Renal histology and apoptosis in ischemia-reperfusion (IR) kidney (WT-S, wild type sham; WT-IRI, IR renal injury in wild-type mice; fat-1, fat-1 induction sham; fat-1 IRI, IR renal injury in fat-1 induction mice). (**A**) Representative kidney section stained peroxidic acid-schiff (PAS) stain; yellow arrows indicate necrotized tubules or the formation of cast Yellow arrowheads indicate loss of brush border or dilated tubules. Black arrows indicate necrotized tubules and inflammatory cells. Original magnification, 200×. Scale bar = 200 μm; (**B**) A semi-quantitative analysis of tubular injury in wild-type and fat-1induction mice kidneys 24 h after IR renal injury (*n* = 5/each group); (**C**) Representative kidney section with H&E stain; yellow arrows indicate necrotized tubules. Yellow arrowhead indicate inflammatory cells. Black arrows indicate necrotized tubules and inflammatory cells. Original magnification, 200×. Scale bar = 200 μm; (**D**) A semi-quantitative analysis of tubule interstitial injury in wild-type and fat-1 induction mice kidneys 24 h after IR renal injury (*n* = 5/each group); (**E**) Representative TUNEL stain of kidney section. Original magnification, 400×. Quantification of Apoptosis Index. Scale bar = 50 μm; (**F**) Representative western blot and quantification of densitometry of caspase-3. * *p* < 0.05 vs. WT-sham kidney, # *p* < 0.05 vs. fat-1-sham kidney, ** *p* < 0.05 vs. WT-IR kidney. Bar represents mean ± S.D.

To evaluate renal tissue damage, hematoxyline and eosin (H&E) and peroxidic acid-schiff (PAS) staining were performed. In H&E-stained cells, tubular cell necrosis, flattening, and interstitial inflammation were observed in wt IRI mice. Tubulointerstitial injury scores were significantly higher in wt IRI mice than in wt sham mice. Fat-1 IRI mice had significantly lower tubulointerstitial injury scores than wt IRI mice (Figure 2A,B). In PAS-stained cells, tubular necrosis, vacuolization, flattening, and loss of brush borders were observed in wt IRI mice. Fat-1 IRI mice had significantly lower tubular injury scores than wt IRI mice (Figure 2C,D). Further examination of renal tissues using the terminal deoxynucleotidyl transferase dUTP nick end labeling (TUNEL) assay demonstrated that fat-1 tubular cells did not undergo apoptosis with IR (Figure 2E). Western blot results showed that the levels of the apoptotic marker cleaved caspase-3 were significantly higher in wt mice after IRI than in wt sham mice. However, renal levels of cleaved caspase-3 were significantly lower in fat-1 IRI mice versus wt IRI mice. Although fat-1 IRI mice had slightly higher cleaved caspase-3 levels than fat-1 sham mice, the difference was not significant (Figure 2F). These results suggest that the enrichment of ω3-PUFAs may function as a negative regulator of apoptosis and may protect renal cells from death induced by IRI.

### 2.3. Effects of Endogenous ω3-Polyunsaturated Fatty Acids on Oxidative Stress

**Figure 3:**
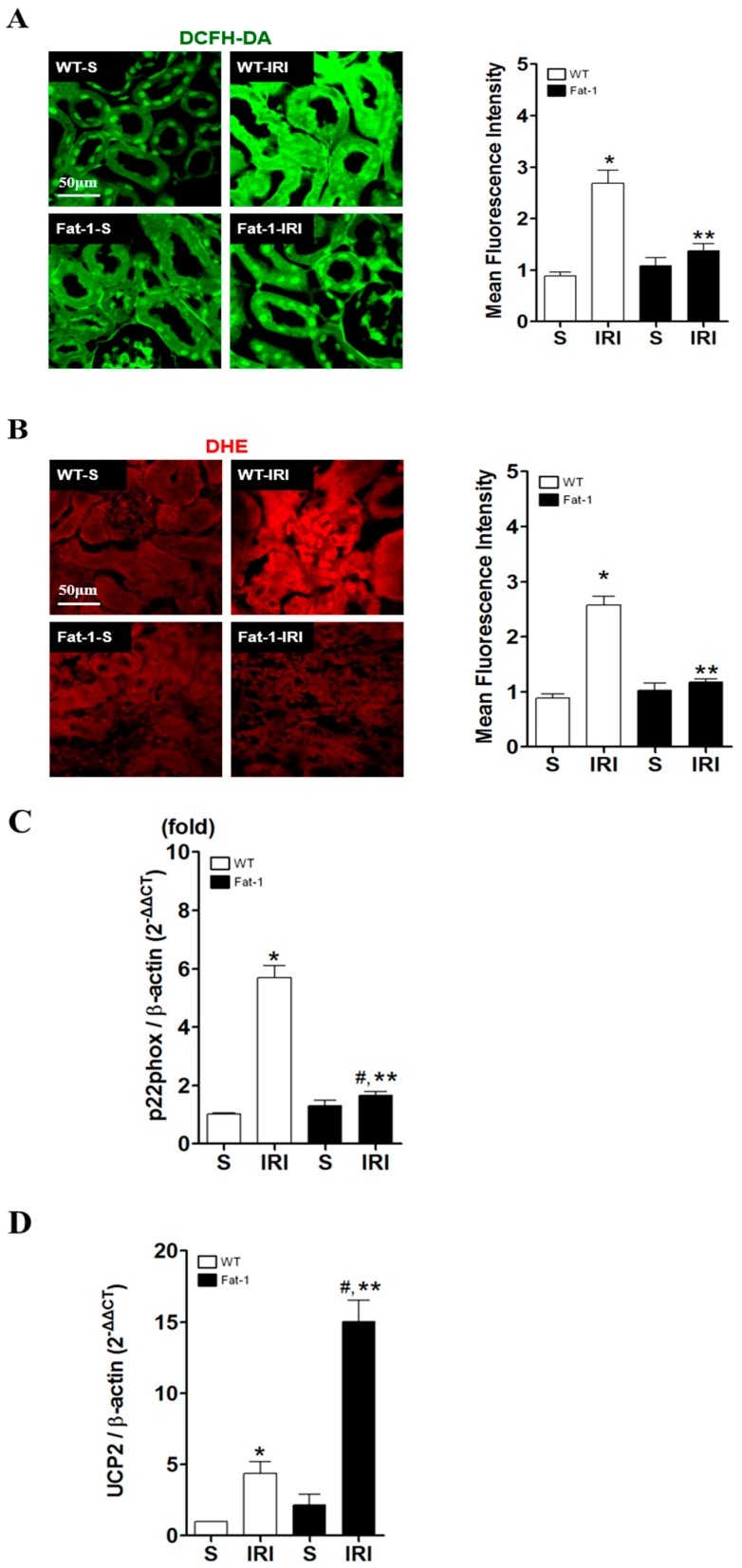

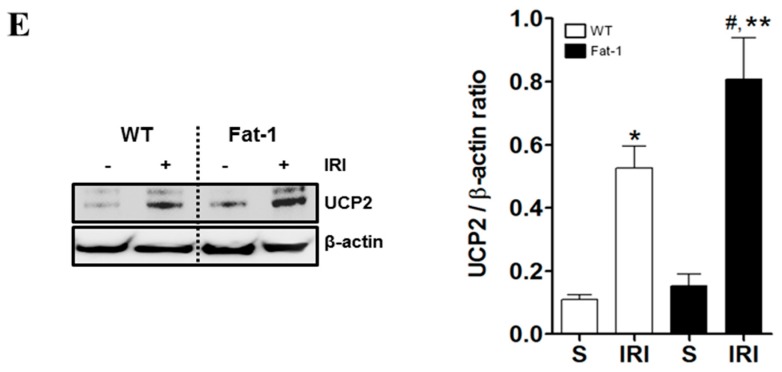
Oxidative stress in ischemia-reperfusion (IR) kidney showing the representative kidney section of reactive oxygen species (ROS) generation in mouse (WT-S, wild type sham; WT-IRI, IR renal injury in wild-type mice; fat-1, fat-1 induction sham; and, fat-1 ischemia-reperfusion injury (IRI), IR renal injury in fat-1 induction mice). (**A**) DCFH-DA staining, detection of H_2_O_2_. Scale bar = 50 μm; (**B**) Docosahexaenoic acid (DHA) staining, detection of O_2_ production. Fat-1 IR mice kidney showed decreased fluorescence intensity of DCFH-DA and DHA compared to wild type IR mice kidney. Scale bar = 50 μm; (**C**,**D**) Real-time polymerase chain reaction (PCR) detection of p22phox and UCP2 mRNA expression in mouse tissues. Data were normalized using β-actin mRNA. Each bar graph, quantification; and (**E**) Evaluation of anti-UCP2 antibody by immunoblot analysis. Left panel: whole tissue lysates from wild and transgenic fat-1 mice were electrophoresed and immunoblotted with anti-UCP2 antibody. Right panel: quantification of densitometry. Data were normalized using β-actin. * *p* < 0.05 vs. WT-sham kidney, # *p* < 0.05 vs. fat-1-sham kidney, ** *p* < 0.05 vs. WT-IR kidney. Bar represents mean ± S.D.

Although ischemic events alone may lead to tubular cell necrosis in the kidney, reperfusion, occurring upon the restoration of blood flow, is associated with the production of ROS, which is considered to play an important role in reperfusion injury [34]. In our study, after verifying oxidative stress in proximal tubules under IR-induced conditions, ROS formation was measured with 2′,7′-dichlorofluorescein diacetate (DCFH-DA) (Figure 3A) and dihydroethidium (DHE) fluorescence (Figure 3B), as reliable markers of ROS in the kidney. ROS were detected in some proximal tubular cells of wt renal tissues during IRI, although this was barely apparent in fat-1 renal tissues. ROS production was significantly greater in wt IRI kidneys than in the control group. However, there was significantly lower fluorescence intensity in the kidneys of fat-1 IRI mice than in wt IRI mice. Although fluorescence intensities were slightly higher in fat-1 IRI mice than in fat-1 sham mice, there were no significant differences in the levels of cleaved caspase-3 between these two groups. The role of ω3-PUFA was determined in terms of ROS-scavenging effects in fat-1 mice via the downregulation of p22phox (Figure 3C). The mitochondrial anion carrier protein uncoupling protein 2 (UCP2) was key in modulating ROS production and inflammatory responses [35]; hence, an increase in UCP2 may act as a protective response against oxidative stress, limiting the production of ROS. As shown in Figure 3D, in response to IRI, fat-1 kidney tissues showed an upregulation of mitochondrial UCP2; as a result, p22phox expression subsequently decreased. Thus, ω3-PUFAs appeared to be involved in the regulation of UCP2, and p22phox expression resulted in enhanced antioxidant effects.

### 2.4. Effects of Endogenous ω3-Polyunsaturated Fatty Acids on Autophagy Activation

**Figure 4:**
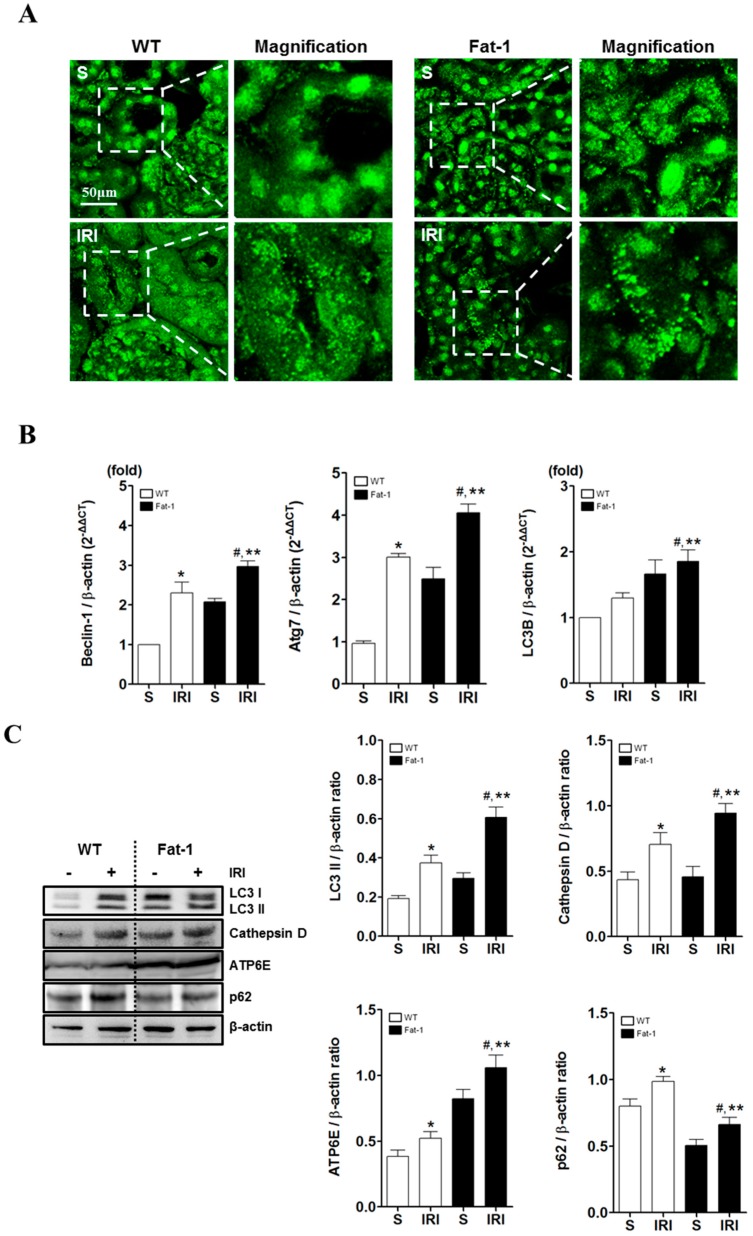

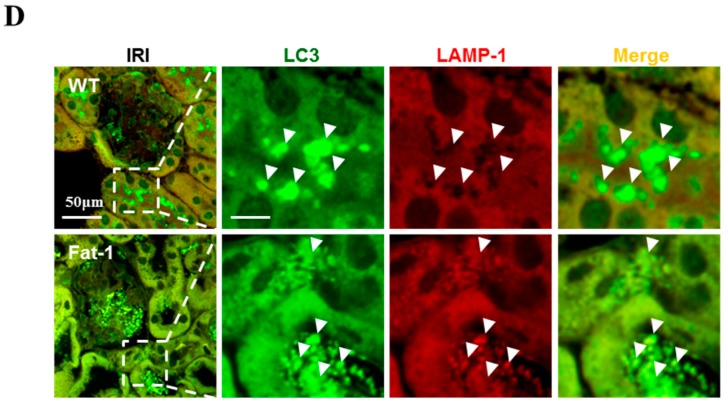
Autophagy activation in IR kidney. ω3--Polyunsaturated fatty acids (PUFA) increased autophagy activation in fat-1 mice. (**A**) ω3-PUFA increases LC3-II accumulation and the appearance of LC3-II punctate. Scale bar = 50 μm; (**B**) The total RNA of tissue following individual experiments was isolated and subjected to real-time PCR. Transcripts of Beclin-1, Atg7, and LC3B were examined after optimization of PCR conditions. Relative mRNA ratios of each autophagy-related gene are described relative to levels of β-actin; (**C**) Tissue proteins were immunoblotted with anti-LC3-II, anti-cathepsin D, ATP6E, and anti-p62. Immunoblotting with β-actin was performed as a loading control. The right bar graph indicates the ratios of LC3-II, cathepsin D, ATP6E, and p62/β-actin as determined by densitometry; and (**D**) Wild and transgenic fat-1 mice were analyzed by fluorescent staining with LC3-II and LAMP-1 to evaluate colocalization of LAMP1-positive lysosomes with LC3-II-labelled autophagosomes. White triangle indicate LC3-LAMP1 colocalized region. The colocalization of punctate LC3-II and LAMP 1 was assayed using confocal microscopy. Cell nucleus was stained with Hoechst (blue). Scale bar = 50 μm. * *p* < 0.05 vs. WT-sham kidney, # *p* < 0.05 vs. fat-1-sham kidney, ** *p* < 0.05 vs. WT-IR kidney. Bar represents mean ± S.D.

A potential role of autophagy in kidney function and survival has been reported in Refs. [28,30]. Progressive autophagy dysfunction can stimulate apoptosis and degeneration in renal cells in IR-induced acute kidney injury (AKI) animals [7]. Thus, the enrichment effects of ω3-PUFAs on autophagy were investigated. First, immunofluorescence was examined with anti-LC3-II in autophagy after inducing IRI. As shown in Figure 4A, levels of LC3-II were higher in wt IRI mice when compared to a control group. These results suggest that autophagosome formation increased after reperfusion in IRI-induced AKI wt mice. However, there was significantly more autophagosome formation in fat-1 mice than in wt IRI mice, as confirmed by assessing Beclin-1, Atg7 and LC3B known regulators of autophagy (Figure 4B). Next, the level of sequestosome 1 (SQSTM1)/p62 was examined. p62, one of the best-known autophagic substrates, is widely used as an indicator because it is involved in the dynamic process of the delivery of autophagic substrates to the lysosome and degradation of autophagic substrates inside the lysosome. Therefore, the measurements of p62 may be a more reliable confirmation of autophagic activity than autophagosomes. Based on these results, the enrichment of ω3-PUFAs would seem to enhance the induction and activation of autophagy; however, the most important molecules in the maturation of autophagosomes/endosomes (such as cathepsin D and ATP6E, which are required for autophagosome–lysosome fusion) are essential for autophagosome clearance and autophagic activation [36]. The impact of n-3 fatty acids on autophagy activation in renal tissues was also investigated. The levels of cathepsin D and ATP6E proteins were increased in IRI-induced AKI fat-1 mice, as shown by immunoblotting (Figure 4C). Immunofluorescence staining was performed using lysosomal-associated membrane protein 1 (LAMP1), a lysosomal marker. The colocalization of LC3-II and LAMP-1, induced by autophagy activation, was examined by confocal laser scanning microscopy (LSM 700); and, was higher in IRI-induced AKI fat-1 mice than in wt IRI mice (Figure 4D). Thus, ω3-PUFAs enhanced autophagy activation by increasing ATP6E and cathepsin D.

### 2.5. Effects of Endogenous ω3-Polyunsaturated Fatty Acids on Adenosine Monophosphate-Activated Protein Kinase Signaling

As ω3-PUFAs induce autophagy and renoprotective responses and AMPK activity is regulated by the reversible phosphorylation of Thr172 in the catalytic α-subunit of AMPK, we determined whether AMPK activation by ω3 enrichment involved the inhibition of IR-mediated mTOR activation [37].

**Figure 5:**
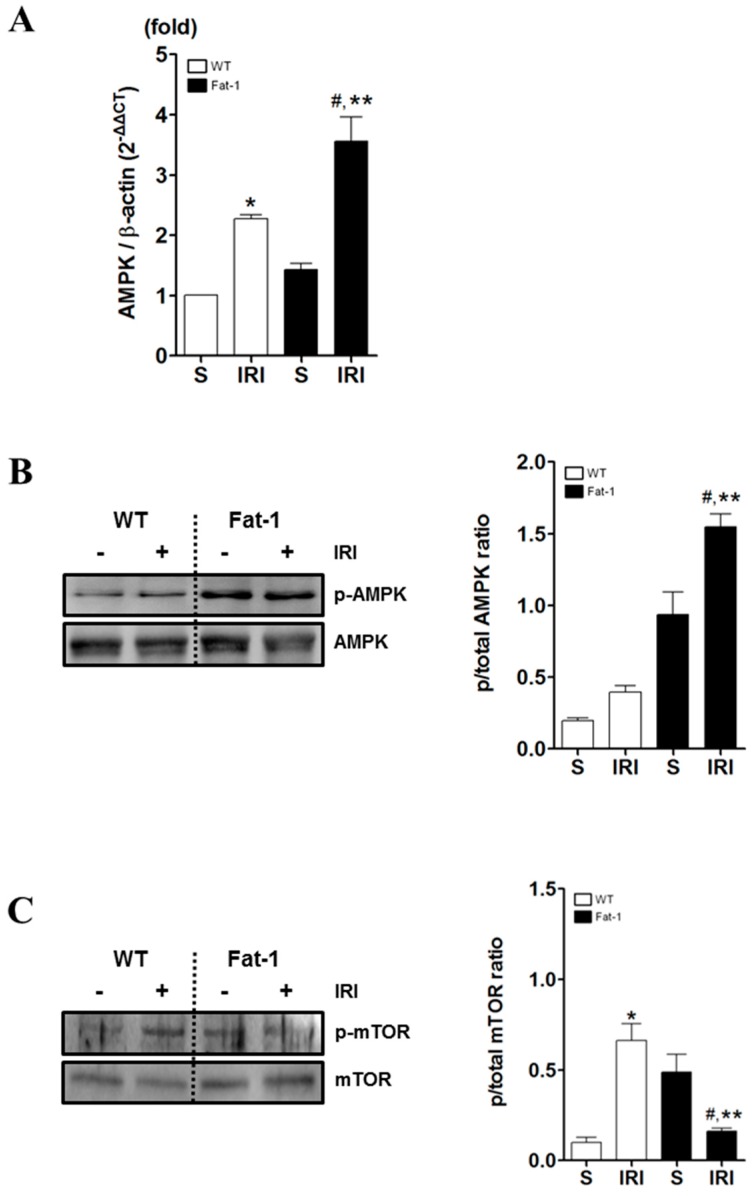
AMP-activated protein kinase (AMPK) and mammalian target of rapamycin (mTOR) expression in IR kidney. Renal expression of AMPK and mTOR. (**A**) Real-time PCR of AMPK in kidney tissue; (**B**) Representative picture and densitometry analysis of western blot of p-AMPK in kidney tissue; and (**C**) Representative picture and densitometry analysis of mTOR in kidney section. Right panel: quantification of densitometry (*n* = 7–8). Data were normalized using β-actin. * *p* < 0.05 vs. WT-sham kidney, # *p* < 0.05 vs. fat-1-sham kidney, ** *p* < 0.05 vs. WT-IR kidney. Bar represents mean ± S.D.

Based on the established role of AMPK in autophagy via the inhibition of mTOR [11], we hypothesized that ω3 enrichment-induced AMPK activation, and subsequent mTOR inhibition, may contribute to the induction of autophagy. To assess this theory, we confirmed the level of AMPK (real-time PCR) and phosphorylation of AMPK at Thr172 (western blot) in IRI (Figure 5A,B). Kidneys were harvested at 12 h (real-time PCR) or 24 h (western blot) after IRI. The level of AMPK and the rate of phosphorylated AMPK expression were higher in fat-1 IRI mice than in wt IRI mice. That is, ω3 enrichment essentially amplified AMPK and phosphorylated AMPK under normal conditions in fat-1 mice.

A previous study showed that IRI in kidneys increased the activation of the mTOR-RPS6KB1/S6K1 (ribosomal protein S6kinase, 70 kDa, polypeptide 1) pathway [38]. Thus, we further examined the effects of ω3-PUFAs on mTOR phosphorylation in kidneys with IRI. In wt IRI mice, it significantly increased the phosphorylation of mTOR at Ser2448 within 24 h when compared to fat-1 IRI mice (Figure 5C). Thus, ω3 enrichment in fat-1 mice decreased the IR induced phosphorylation of mTOR in the kidney. Our findings indicated that ω3-PUFA-induced AMPK activation may overcome mTOR activation during IRI, leading to renoprotective effects.

## 3. Discussion

**Figure 6:**
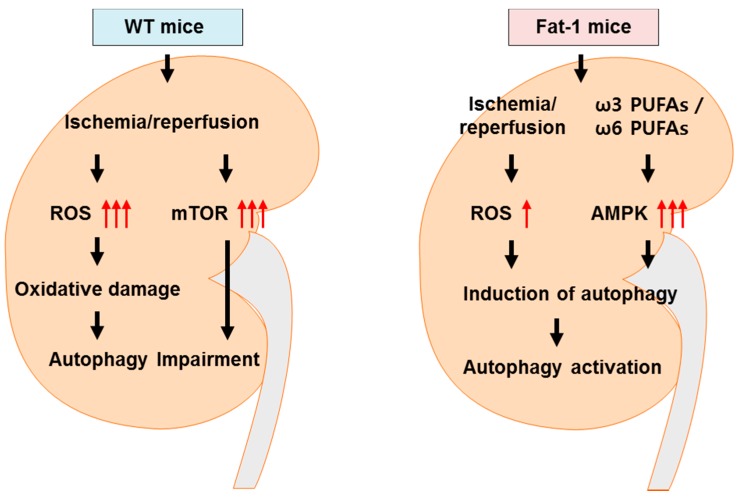
Schematic diagram. Ischemia-reperfusion (IR) increases kidney damage, with increased oxidative stress and less pronounced AMPK activation. Activation of AMPK inhibits reactive oxygen species-induced autophagy impairment and induces autophagy, resulting in preserved adenosnine triphosphate (ATP) content and kidney homeostasis. AMPK-mediated autophagy was reduced in wt mice damaged by IR, prompting kidney dysfunction. However, in fat-1 mice, high ω3-PUFAs increased AMPK-mediated autophagy and protected the kidney against IR via induction of autophagy and enhancement of autophagy activation.

In the present study, we demonstrated that ω3-PUFAs significantly attenuated kidney injury by enhancing autophagy activation and inhibiting apoptosis in a renal IRI model (Figure 6). These results indicated that increasing the levels of endogenously synthesized ω3-PUFAs in the kidney may reduce the risk of IRI and attenuate oxidative stress in fat-1 transgenic mice [24]. This was associated with antioxidant effects. Furthermore, protection from renal injury in fat-1 mice correlated with the formation of UCP2 and p22phox in kidney tissue. Importantly, ω3-PUFAs appear to be involved in a renoprotective mechanism for cell survival.

Oxidative stress plays a critical role in the pathophysiology of IR-induced kidney injury [39]. IRI generates ROS, including hydrogen peroxide and hydroxyl radicals, which are known inducers of lipid peroxidation [40]. In our study, ROS and cleaved caspase-3 levels were only slightly higher in fat-1 IRI mice than in fat-1 sham mice. However, TUNEL, H&E, and PAS staining, and creatinine serum levels were significantly greater in fat-1 IRI mice than in fat-1 sham mice. Although this cannot be fully explained, we suggest that ROS generation and cleaved caspase-3 levels were nearly diminished in fat-1 mice with IR renal injury. However, caspase-independent apoptosis (AIF, Endo G, HtrA2) may also be inhibited to some degree [41]. Thus, fat-1 mice did not show complete inhibition of any renal injury, as evidenced by the TUNEL staining, renal tubulointerstitial injury, and serum creatinine results. Our results suggest that fat-1 can protect against damage from oxidative stress to the kidney through the regulation of UCP2 and p22phox. Accordingly, reduced ROS levels were also observed in the kidneys of fat-1 mice, leading to renoprotective effects.

Although the key regulators, Nrf2 and HO-1 are well known in IRI, and omega-3 treatment induced the activation of Nrf2 and HO-1, resulting in improving the ROS including (UCP2 and NADPH oxidase) [42], recent research has demonstrated the role of ROS in IR-induced autophagy regulation [43,44]. SQSTM1/p62 and polyubiquitinated protein aggregates may be seen as markers of autophagy function. Furthermore, p62 is a key factor that controls cell death versus survival [45] and is an autophagy-related protein normally degraded by lysosomal proteases through an interaction with LC3-II [45]. Its accumulation reflects the inhibition of proteasomal activity [46]. Similarly, diminished p62 levels are associated with autophagy activation. Dysregulation of autophagy can result in renal cell death, as noted in several kidney diseases. In this study, the basal level of LC3-II was greater (leading to autophagy activation) in fat-1 mice than in wt mice after IRI. In addition, our results showed LC3 is increased in sham fat-1 mice. Although there is no clear explanation of elevation of LC3 in fat-1 sham mice kidney, Bak et al reported the increase of LC3 in fat-1 sham mice Purkinje cell [36]. There exists some studies that omega 3 may induce the autophagy in various cells, including macrophage [32,47].

Cathepsin D (a lysosomal aspartic protease) is an effector enzyme of autophagosome degradation in lysosomes, ultimately resulting in the acceleration of autophagic activation. ω3-PUFAs can directly augment cathepsin D expression in the cerebellum [36]; moreover, they can increase lysosome activity and eventually induce proteolytic activation of cathepsin D. In addition, ATP6E expression is increased in fat-1 mice where high levels of cathepsin D and ATP6E in renal cells significantly activate autophagy activation, inducing the formation of acidic autophagic vacuoles and autolysosomes. Thus, ω3-PUFAs may play an important role in renoprotective effects via the activation of autophagy activation.

The energy-sensing LKB1-AMPK pathway regulates autophagy [28]. Although the AMPK may be regulated by other key molecules [48], recent studies have demonstrated that AMPK is the main initiator of stress-triggered autophagy, such as oxidative stress, hypoxia, and nutrient deprivation [11,49]. AMPK is considered an autophagy-promoting kinase, whereas mTOR has been reported to have the opposite effect [49]. mTOR kinase is a major negative regulator of autophagy, and mTOR signaling is frequently dysregulated in cancer, where LKB1/AMPK signaling can act upstream of it [31]. Our data demonstrated that ω3-PUFAs overrode mTOR activation induced by IRI through autophagy activation in fat-1 mice. These findings supported the involvement of AMPK-mTOR signaling as an effector of the autophagy activation pathway in IRI kidneys, leading to renoprotective responses. Our fat-1 sham mice showed higher levels of mTOR than those of the wt sham mice. In our data, it was confusing that mTOR was more elevated in fat-1 sham mice than in wt sham mice, instead of decreased in fat-1 IRI mice than in wt IRI mice. However, ω3-PUFAs increased mTOR-p70s6k signaling, which controls muscle protein anabolism and muscle protein growth in humans [50]. It may be possible that omega-3 elevates mTOR activity in basal status kidney, like muscle.

This study had some limitations. Fatty acid component in the experiments have not been measured and the direct evidence to test the role of ω3-PUFA in the improvement of renal injury are missing. Therefore, we cannot exclude the possibility that a decreased ω-6 may also affect those positive effects. In addition, although we examined autophagy activation and the beneficial effects of ω3-PUFAs in fat-1-overexpressing IRI mice, we did not determine the beneficial effects of medication with ω3-PUFAs in IRI. Moreover, it has not been determined whether ω3-PUFAs have beneficial effects in patients with acute kidney injury.

Taken together, our results highlighted that fat-1 overexpression resulting in ω3-PUFA enrichment helped to prevent IR-induced renal injury. This occurred via the regulation of antioxidant gene expression, blocking ROS production, and repressing oxidative stress as a result of ω3-PUFA-induced autophagy activation in the kidneys of fat-1 mice versus wt mice. ω3-PUFA enrichment reduced renal cell damage, and promoted autophagy activation through cathepsin D-mediated autophagy activation. It is known that cathepsin D reduces oxidative stress-induced cell death via the activation of autophagy [51]. Furthermore, ω3-PUFA enrichment activated ATP6E activity, which significantly participates in renal cell survival. The present study was to investigate the autophagy activity of ω3-PUFAs in the kidney, where basal autophagy in fat-1 mice was activated, and had protective effects on renal cells through autophagy clearance-mediated ATP6E regulation. In addition, we suggest that AMPK activation by ω3-PUFA enrichment may affect overall renoprotective activity and autophagy function. These processes may underlie renal cell survival and may be potential preventive targets for the treatment of IRI related to renal transplantation.

## 4. Materials and Methods

### 4.1. Animals

Dr. Jing Xuan Kang of Harvard Medical School (Boston, MA, USA) provided the fat-1 transgenic mice (10 weeks old, male). Generations of these mice were mated to gain homozygous transgenic and heterozygous mice. All of the transgenic fat-1 mice used were homozygous and male, and the presence or absence of the fat-1 gene in each mouse was confirmed by genotyping. Food and water were freely consumed, and mice were housed in a room maintained with a 12/12 h light/dark cycle. All of the animal experiments were conducted with the approval of the Animal Use and Care Committee at Chungnam National University School of Medicine (CNU-00457, 21 March 2014). The mice were divided into four groups: wt sham (*n* = 10), fat-1 sham (*n* = 10), wt IR (*n* = 15), and fat-1 IR (*n* = 15). IR injury was performed as described previously [43]. Mice were treated and sacrificed at 10:00 am each day. Mice were anesthetized with an intraperitoneal injection of ketamine (60 mg/kg body mass) and xylazine (8 mg/kg). After an abdominal incision, both renal pedicles were clamped bluntly. During the procedure, a heating pad was used to maintain the mouse’s body temperature at 35–36 °C. After 35 min of ischemia, the clamps were removed. Only the wt sham model received surgical procedures without clamping. Blood and kidney tissues were collected.

### 4.2. Blood and Tissue Preparation

Renal function was evaluated as described previously in Reference [2]. Blood was obtained from the inferior vena cava of the anesthetized mice. The blood was placed in microcentrifuge tubes (4 °C) and for BUN and creatinine serum, aliquots of serum were analyzed using a chemistry auto-analyzer, Toshiba 200FR (Toshiba Medical Systems Co., Tokyo, Japan). The tissues were prepared as described previously in Reference [2]. Briefly, the left kidney was removed immediately after sacrifice and cut into three pieces. Two pieces were snap-frozen in liquid nitrogen and stored at 70 °C until the extraction of RNA and protein analysis. The other piece of kidney was fixed in 10% buffered formaldehyde at room temperature (RT) and then embedded in Paraplast (Sherwood Medical, St. Louis, MO, USA) for light microscopy.

### 4.3. Tissue Injury Score

The kidney tissue was made into paraffin blocks, cut into 4 μm, and attached to a slide glass. The sections were deparaffinized with xylene, stained with H&E and PAS, and examined under a microscope (Olympus BX51, Olympus, Tokyo, Japan). Five consecutive fields were examined at ×200 magnification and tissue injury scores were averaged per slide. For PAS staining, tubular necrosis was defined as the loss of intra-luminal aggregation of cells and proteins, or proximal tubular brush border blebbing of apical membranes. Tubular necrosis (injury) in PAS-stained sections was scored as follows: 0: normal, 1: <10%, 2: 10–25%, 3: 26–75%, and 4: >75% For the H&E sections, renal cortical vacuolization, proximal tubule simplification, renal cortical vacuolization, and peritubular/proximal tubule leukocyte infiltration were evaluated and scored as follows: normal 0, mild injury 1, moderate injury 2, and severe injury 3. The injury scoring in PAS and H&E stain were evaluated by an experienced pathologist in a blind fashion.

### 4.4. Confocal Microscopy

Levels of LC3-II and LAMP-1 were observed by confocal microscopy after immunofluorescent staining. The cells were incubated with primary antibody against LC3-II (1:400, Medical & Biologucal Laboratory Co., Ltd., Woods Hole, MA, USA) and LAMP-1 (1:400, Santa Cruz Biotechnology, Dallas, TX, USA). The sections were washed and the secondary antibody incubated at RT for 1 h. After washing, nuclei were stained with 4′,6-diamidino-2-Phenylindole (DAPI) for 5 min and then mounted. A fluorescence image was obtained using a confocal microscope (LSM 700; Zeiss, Jena, Germany).

### 4.5. Western Blot Analysis

Tissues were lysed in 1 mL ice-cold PRO-PREP buffer (iNtRON, Seongnam, Korea). Protein concentrations in supernatants were evaluated with a BCA protein assay kit (Thermo Scientific, South Logan, UT, USA). Protein (30 μg/lane) was electrophoresed on 10–15% SDS gel, and then transferred to polyvinylidene fluoride (PVDF) membranes. Membranes were blocked with 5% non-fat dry milk for 2 h at RT and then incubated with rabbit primary antibodies against β-actin (1:1000, Santa Cruz Biotechnology), LC3-II, and p62 (1:1000 and 1:1000, respectively, Sigma-Aldrich, St. Louis, MO, USA), ATP6E, cathepsin D, and caspase-3 (1:1000, 1:1000 and 1:500, respectively, Cell Signaling Technology, Danvers, MA, USA) at 4 °C overnight. Membranes were incubated with HRP-conjugated anti-rabbit IgG secondary antibodies (1:1000, Abfrontier Co., Ltd., Seoul, Korea) for 2 h at RT. The protein bands were visualized using a chemiluminescence detection kit (Thermo Scientific, South Logan, UT, USA). The same membranes were subsequently used for β-actin immune detection, and equal protein loading was ensured. The optical density for quantification was obtained using Gel-Pro Analyzer version 3.1 (Media Cybernetics, Silver Spring, MD, USA).

### 4.6. Measurement of Reactive Oxygen Species Production

ROS levels were measured using DCFH-DA, 10 μM (Molecular Probes, Inc., Eugene, OR, USA) and DHE, 10 μM (Molecular Probes, Inc., Eugene, OR, USA), as described previously in Reference [52]. Tissues were incubated for 60 min in Krebs-HEPES buffer containing DCFH-DA and DHE and washed twice. After mounting, fluorescence images were acquired with confocal microscopy.

### 4.7. Real-Time Polymerase Chain Reaction

The RNA was extracted from kidney using an RNeasy Mini Kit (Qiagen, Hilden, Germany) according to the manufacturer’s recommendation. cDNA was synthesized from 2 µg total RNA using an oligo dT primer (Amersham Pharmacia), deoxynucleotide tirphosphates (Amersham Pharmacia, Piscataway, NJ, USA), moloney murine leukemia virus reverse transcriptase (Gibco-BRL, Grand Island, NY, USA), 0.1 M dithiothreitol, and buffers in a volume of 20 µL. The cDNA reaction mix was diluted to a total volume of 40 µL and PCR was performed to amplify the following specific cDNAs: (p22phox; primers: sense 5′-GTG GAC TCC CAT TGA GCC TA-3′; antisense 5′-CTC CTC TTC ACC CTC ACT CG-3′); UCP2; primers: sense 5′-GCG TTC TGG CCA TCC TA-3′; antisense 5′-GCT CTG AGC CCT TGG TGT AG-3′; Beclin-1; primers: sense 5′-GGC CAA TAA GAT GGG TCT GA-3′; antisense 5′-GCT GCA CAC AGT CCA GAA AA-3′; Atg7; primers: sense 5′-TCC GTT GAA GTC CTC TGC TT-3′; antisense 5′-CCA CTG AGG TTC ACC ATC CT-3′; *LC3B*; primers: sense 5′-CGG CTT CCT GTA CAT GGT TT-3′; antisense 5′-ATG TGG GTG CCT ACG TTC TC-3′; AMPK; primers: sense 5′-CTC CCA GTT ATC GAC CCA GA-3′; antisense 5′-GCT TGG GGA ACT CGA TGA TA-3′; β-actin; primers: sense 5′-TGT TAC CAA CTG GGA CGA CA-3′; and, antisense 5′-GGG GTG TTG AAG GTC TCA AA-3′). PCR was carried out using SYBR Green PCR mastermix (Qiagen). The amplification was performed in 20 µL reaction volumes consisting of 10 µL iQ SYBR Green PCR mastermix, 2 µL primers, 2 µL cDNA, and 6 µL water. Amplification and detection were performed using a thermal cycler (Rotor-Gene 6000, Corbett Research, Mortlake, Australia). SYBR green fluorescence was measured at the end of each cycle using the comparative threshold cycle (*C*_t_) method: 2^–^^ΔΔ*C*t^ = 2^–[(*C*t of target gene−*C*t of β-actin in treated mice)−(*C*t of target gene−*C*t of β-actin in sham mouse)]^.

### 4.8. Terminal Deoxynucleotidyl Transferase dUTP Nick End Labeling Staining

Paraffin sections were permeabilized, and rehydrated using proteinase K (20 mg/mL in PBS) for 15 min at RT. Endogenous peroxidase was blocked with 3% hydrogen peroxide diluted in PBS. Sections were experimented for TUNEL staining using the ApopTag Peroxidase in Situ Apoptosis Detection Kit (Chemicon International, Temecula, CA, USA) as per the manufacturer’s recommendations. TUNEL-positive cells were identified with fluorescent signals using an LSM 5 Pascal Exciter confocal microscope. To evaluate apoptosis semi-quantitatively, five microscopic fields in microscopic section were selected randomly at ×200 magnification. The apoptosis index (number of TUNEL positive cells/DAPI positive cells) was calculated using the Image-Pro Plus 6.0 (Media Cybernetics, Silver Spring, MD, USA).

### 4.9. Statistical Analysis

Data were expressed as means ± SDs. Multiple comparisons among groups were analyzed using one-way ANOVA with a post hoc Bonferroni correction. We used SPSS software (ver. 11.0 for Windows; SPSS, Inc., Chicago, IL, USA). Differences between groups were considered significant at *p* < 0.05.

## 5. Conclusions

In conclusion, this study demonstrated the renoprotective effects of ω3-PUFAs by reducing ROS and regulating antioxidant gene expression in renal cells. The renoprotective mechanism of these effects in fat-1 mice was likely to be related to the AMPK-mediated inhibition of phosphorylation of the mTOR and autophagy activation by an increase of ATP6E and cathepsin D, indicating enhanced autophagy activation. Thus, the present study clearly indicated that ω3-PUFAs are candidates for renal protective agents in AKI.

## Abbreviations

IRI	Ischemia-reperfusion Injury
ω3-PUFAs	ω3-Polyunsaturated fatty acids
AKI	Acute kidney injury
WT	Wild-type
mTOR	Mammalian target of rapamycin
AMPK	AMP-activated protein kinase
ROS	Reactive oxygen species
UCP2	Uncoupling protein 2
LC3	Microtubule-associated protein 1A/1B-light chain
SQSTM1	Sequestosome 1
